# Coral larvae have unique transcriptomic responses to pathogenic and probiotic bacteria

**DOI:** 10.1007/s00338-026-02830-1

**Published:** 2026-02-24

**Authors:** Erin M. Borbee, Isabella V. Changsut, Kira Bernabe, Alicia Schickle, David Nelson, Koty H. Sharp, Lauren E. Fuess

**Affiliations:** 1 Texas State University, San Marcos, TX 78666 USA; 2 Southwestern University, Georgetown, TX 78626 USA; 3 University of Rhode Island, Kingston, RI 02886 USA; 4 University of Louisville, Louisville, KY 40292 USA; 5 Roger Williams University, Bristol, RI 02809 USA

**Keywords:** Larvae, Immunity, Transcriptomics, Probiont, Pathogen

## Abstract

The growing prevalence of coral disease and other stressors threatens coral reefs, and evidence suggests these stressors not only affect adult corals but may also have impacts on early life stages. Yet, our understanding of coral larval physiology, including immunological function, is significantly limited. Here, we leverage the temperate coral experimental system *Astrangia poculata* to investigate the transcript-level response of *A. poculata* to exposure to pathogenic and probiotic bacteria. The bacteria tested in the experiment were the known coral pathogen (*Vibrio coralliilyticus* RE22) and a probiont (*Phaeobacter inhibens* S4) previously demonstrated to inhibit the pathogen’s growth. In this study, we aim to (1) characterize the general immune response of *A. poculata* larvae to bacterial stimuli, (2) identify defense mechanisms that may aid in host resistance to pathogens, and (3) characterize the impacts of probiont exposure on *A. poculata* larval gene expression. Our data identify both shared and divergent larval immune responses to probiotic and pathogenic bacteria. Immunological peptidases were enriched in response to bacteria—expression of endopeptidases increased in response to probiont and pathogen, and metallopeptidase activity was enriched in response to the pathogen. Clustering analyses further revealed a diverse array of immune components in response to the pathogen, while its response to the probiont was associated with a broad range of developmental and metabolic terms. Collectively, the data from this study identify immune components that may play a role in coral response to bacteria during their early life stages, with broad relevance to current restoration and conservation approaches.

**Supplementary Information:**

The online version contains supplementary material available at 10.1007/s00338-026-02830-1.

## Introduction

Corals are facing major declines globally due to several environmental stressors including increased sea surface temperatures, pollution, and disease (Hoegh-Guldberg 1999; Hoegh-Guldberg et al. 2017; Alvarez-Filip et al. 2009; De’ath et al. 2012). Coral diseases, including stony coral tissue loss disease (SCTLD), black band disease, yellow band disease, and white plague disease, have been estimated to collectively contribute to 50–60% of coral mortality in parts of the Caribbean and continue to increase in frequency and severity across tropical reef ecosystems (J. Miller et al. 2009; Alvarez-Filip et al. 2022). The increasing threat posed by disease to corals has led to a growing field of research aimed at understanding the mechanisms underlying the coral immune response. Corals possess a complex innate immune system that enables defense against stressors including pathogens, oxidative stress, and UV stress (Mydlarz et al. 2006, 2016; Parisi et al. 2020). The coral immune response begins with pathogen recognition, followed by signaling pathways that lead to effector responses (Mydlarz et al. 2016). The pattern recognition receptors (PRRs) in the coral immune system include toll-like receptors (TLR), NOD-like receptors, and lectins (Palmer et al. 2012; Traylor-Knowles and Connelly 2017). These molecules bind to highly conserved structural components on the surface of microbes called microbe-associated molecular patterns (MAMPs) (Koropatnick et al. 2004). These patterns include flagellin, lipopolysaccharide (LPS), and more (Toledo-Hernandez and Ruiz-Diaz 2014). Once detected by PRRs, the receptors trigger signaling pathways (i.e., TLR pathways, complement pathway, cell activation, and protease pathways) leading to a diverse array of effector responses (Palmer and Traylor-Knowles 2012). These responses include antioxidant response (catalase, peroxidase, etc.) (Armoza-Zvuloni and Shaked 2014; Krueger et al. 2015), activation of melanin synthesis (cytotoxicity, pathogen encapsulation, UV shading) (Palmer et al. 2008, 2012; Petes et al. 2003), and production of antimicrobial compounds (Vidal-Dupiol et al. 2011; Mason 2021).

While knowledge of coral immunity and response to disease has dramatically increased in recent decades, most research has focused on adult corals leaving the understanding of larval pathogen responses and immunity lagging behind. Although data suggest that bacterial pathogens can reduce larval recruitment and settlement success (Edmunds 2000), the interactions between coral larvae and bacteria, including potential larval immune/defense responses, remain largely uncharacterized. Data from a variety of marine invertebrate taxa demonstrate that early life stages often lack a mature allorecognition system, or system responsible for identifying non-self-cells/tissue, limiting their ability to mount an immune response (Puill-Stephan et al. 2012). Among cnidarian orders, there is great variation in rate of allorecognition system development. In one hydrozoan (*Hydractinia symbiolongicarpus*), allorecognition systems are first detected at two weeks (Wilson and Grosberg 2004), whereas in some scleractinian coral species (*Stylophora pistillata* and *Seriatopora *spp.), these systems appear around four months into development (Nozawa and Loya 2005; Frank et al. 1997). Understanding of development of other immune components in corals is similarly limited. Some studies have identified the presence and activity of immune components involved in the melanin and antimicrobial pathways in lecithotrophic coral larvae prior to settlement, though timing of onset of these responses is unknown (Palmer et al. 2012). While these studies point toward the existence of some immunological capabilities in coral larvae, a comprehensive model including composition, function, and activity would help predict response to the increasing threat of pathogen exposure. Specifically, we lack knowledge regarding how these immune components may protect larvae against potential environmental pathogens.

Beyond the need for understanding coral larval pathogen responses, improved understanding of reef-building coral immunity during early life stages is also essential for informing effective restoration approaches, especially the use of probionts (beneficial microbes) to increase coral larval and post-settlement survival (Reshef et al. 2006). Probiotic methods have primarily been applied to adult corals and aim to manipulate the hosts’ microbial communities associated to include beneficial microorganisms, allowing corals to adapt to rapid environmental changes (Voolstra and Ziegler 2020; Thatcher et al. 2022). Probiotic applications in corals have demonstrated benefits on skeletal structure (Moradi et al. 2023), resistance to bleaching (Rosado et al. 2019), and in slowing disease progression (Ushijima et al. 2023). Because microbiomes in juveniles of multiple scleractinian coral species are much more diverse and dynamic (Lema et al. 2014; Epstein et al. 2019), juvenile microbiomes are likely highly manipulable by early probiotic intervention and therefore could be a powerful tool for restoration. However, knowledge of the impacts of probiotics on larval and adult survival is limited to a handful of studies, one of which found that probiotics had little impact on coral recruit resistance to stony coral tissue loss disease (SCTLD) exposure (Demko et al. 2025). Ultimately, the dynamic nature of the microbiome in early life stages may make it ideal for manipulation and restoration practices, but a comprehensive understanding of how introduction of microbes at early life stages impacts the host, its development, and its microbiome is essential for evaluating the potential efficacy of these approaches.

Here, we aim to gain insight into the coral larval immune response using the coral *Astrangia poculata*. *Astrangia poculata* is a temperate stony coral native to the east coast of the USA with well-established spawning and larval rearing methods in a laboratory setting. This species of coral acquires its algal symbionts horizontally from the environment rather than from its parent, meaning that larvae lack algal symbionts during initial development. Leveraging existing spawning techniques for *A. poculata* and experimental exposures to pathogenic and probiotic bacteria, we aim to: (1) characterize the general immune response of *A. poculata* larvae to bacterial stimuli, (2) identify putative defense mechanisms which may facilitate *A. poculata* larval resistance to pathogens, and (3) characterize the transcript-level responses of *A. poculata* larvae to a probiont. The data we present here enhance our understanding of early life stage immunity in corals by revealing unique transcriptomic responses of *A. poculata* larvae to pathogenic and probiotic bacterial exposures and generate new hypotheses on the impacts of bacterial exposure during early life stages, with broad relevance for current restoration and conservation applications.

## Methods

### Coral collection, spawning, and husbandry

*Astrangia poculata* colonies were collected from Fort Wetherill State Park in Jamestown, RI, in late July–early August of 2021. Corals were collected by SCUBA using a hammer and chisel at a depth of 4.5-8m. The colonies collected ranged in size from 2 to 5cm^2^ in surface area and in algal symbiont density from brown (high symbiont density), white (low symbiont density), and mixed (coral containing polyps of high and low symbiont density). At the surface, corals were cleaned to remove algae and invertebrates like sponges or worms living on the coral. Once cleaned, corals were placed in 1 µm filtered seawater and transported back to the marine laboratory at Roger Williams University in coolers.

At the laboratory, corals were placed in raceways of seawater held at 22 °C and monitored for an hour. To induce spawn, corals were removed from the raceway and put in 4L containers with filtered seawater at 27 °C and no flow. Because *A. poculata* is gonochoristic (colonies contain only eggs or sperm, not both), 5–6 colonies were placed in each container to increase the chances of getting eggs and sperm in the same container. Once gamete release was documented in each container, the gametes were transferred to a clean container to allow for fertilization. In the case that multiple containers released gametes at the same time (within 30 min of each other), the gametes from those containers were pooled and used for monitoring development.

Fertilization was confirmed by the observance of the first cell division, and embryonic development was monitored for the first 24 h according to a previously determined timeline (Szmant-Froelich et al. 1980). After 24 h, swimming planulae were present and moved into plastic containers with 53 µm mesh bottoms and placed in the 22 ºC seawater raceways with flow to flush out any remaining sperm and debris. Larvae continued to grow and develop up to 72 h post-fertilization, at which point they had fully developed apical cilial tufts, a morphological signal that larvae had fully developed and were prepared to settle.

### Experimental setup

Approximately 96 h post-fertilization, larvae were aliquoted into 10 mL seawater vials (n = 100 larvae per vial; 15 vials total). Each vial was later transferred to a sterile petri dish for experimental setup. An additional subset of dishes (2 dishes per treatment) was set up with the same number of larvae and same treatments to monitor for survival past our collection timepoints for RNA. Each dish was then randomly assigned to one of three treatments: control (0.2 µm filter sterilized seawater), probiont (*Phaeobacter inhibens* S4 (Takyi et al. 2023)), or pathogen (*Vibrio coralliilyticus* RE22Sm (Spinard et al. 2015); n = 5 replicates per treatment). *Vibrio coralliilyticus* RE22Sm was chosen as the pathogen strain as it is a known coral pathogen which has been used in previous *A. poculata* pathogen exposure experiments (Awkerman 2021). *Phaeobacter inhibens* S4 was chosen as the probiotic due to its demonstrated effects providing protection against *V. coralliilyticus* RE22Sm infection in aquaculture settings in other marine invertebrate species (Modak and Gomez-Chiarri 2020). For both bacterial treatments, bacteria were first streaked from a glycerol stock and then grown up overnight in MYP30 broth in a shaking incubator at 27 °C and 200 rpm. Bacteria were then quantified with a UV–Vis spectrophotometer (OD_600_) using a previously generated growth curve for each strain. Once at stationary phase, the bacteria were pelleted using centrifugation for 5 min at 3,000×g at 4 °C and washed three times with 0.2 µm filter sterile seawater (FSW). Bacterial cell concentration was calculated again (OD_600_) to obtain an accurate concentration after washes, prior to addition to the treatment dishes. The bacterial cultures were then diluted to the same concentrations using FSW and were added to each dish in a volume that achieved a final concentration of 10^8^ cells/mL in each dish. Once the volume of bacteria was determined for the treatment dishes, the same volume of FSW was added to each control dish as a placebo treatment. All dishes were incubated at room temperature with gentle shaking, and after four hours, larvae were picked out of the dishes using Pasteur pipets and placed in 2 mL cryovials (1 dish of 100 larvae per vial). Following addition of larvae, a drop of TRIzol reagent (Thermo Fisher Scientific) was added to each tube to kill the larvae. Samples were spun down for 2 min at 13,000×g and as much liquid as possible was removed using a pipette without disturbing the pellet. After spinning and removing supernatant, 1 mL of TRIzol reagent was added to each tube to preserve the larvae. Samples were then vortexed vigorously for 30 s to lyse cells and then stored at − 80 °C until RNA extraction at a later date.

### RNA extraction and sequencing

RNA was extracted from the larval samples following the Thermo Fisher Phasemaker tube extraction protocol with modifications from the Babonis Lab at Cornell. Briefly, TRIzol samples were thawed on ice until just melted. While waiting for samples to thaw, two Phasemaker tubes (Thermo Fisher Scientific) per sample were spun at max speed (16,000×g) for 1 min. After thawing, each sample was transferred into the first of the two Phasemaker tubes on ice. Next, 200 µL of chloroform per mL of TRIzol was added to each sample. Samples were manually shaken vigorously for 15 s to mix the chloroform and TRIzol reagent and then incubated on ice for 10 min. Samples were then spun down at 16,000×g for 15 min at 4 °C. The upper clear aqueous phase (~ 600 µL) was transferred to a second Phasemaker tube, and 600 µL of phenol/chloroform/isoamyl alcohol (25:24:1) was added to the sample, being sure to draw from the bottom organic phase of the mixture. Samples were again manually shaken for 15 s to mix layers well and then were incubated on ice for 5 min. Samples were centrifuged at 16,000×g at 4 °C for 15 min to allow for phase separation. The RNA was contained in the upper clear aqueous layer which was then transferred into a clean 1.5 mL tube. Following extraction, 1 µL of glycogen was added to each sample to help with pelleting the RNA for washes. RNA was pelleted in each sample by adding 500 µL of RNase-free isopropanol to each sample, shaking for 15 s, incubating at room temperature for 15 min, and then spinning down at 16,000×g at 4 °C for 15 min. Liquid was carefully removed from each sample being sure not to disturb the pellet. The RNA pellet was washed twice by adding 1 mL of ice cold 75% ethanol to each tube and vortexing to loosen the pellet. After each wash, samples were spun down at 16,000×g at 4 °C for 5 min and ethanol was removed. After the last wash, as much liquid as possible was removed from the tube without disturbing the pellet, and the pellet was air-dried for 5–10 min at room temperature. The final pellet was resuspended in RNase-free water and incubated on a heat block at 55 °C for 15 min to better solubilize the RNA.

Following extraction, quality and quantity of the RNA was checked using a spectrophotometer (Biotek Cytation 1). Samples were then diluted to the same concentration (10 ng/µL) and submitted to the University of Texas at Austin Genomic Sequencing and Analysis Facility for library prep and sequencing for TagSeq (Lohman et al. 2016). Samples were sequenced on the Illumina NovaSeq 6000 SR100 on a 1 × 100 bp run with normal coverage (3–5 million reads per sample).

### Bioinformatics

Sequence data were processed following an established pipeline from Dr. Misha Matz at UT Austin (https://github.com/z0on/tag-based_RNAseq) (Meyer et al. 2011). This pipeline begins by trimming adapters and low-quality bases from reads using Cutadapt (Martin 2011) and deduplicating reads using a custom perl script. Next, reads were mapped to an *A. poculata* reference transcriptome using Bowtie2 (Langmead and Salzberg 2012). Finally, we generated counts of each transcript per sample and assembled a count matrix that was used as input for downstream analyses.

### Differential expression

The count matrix generated above was analyzed with DESeq2 to determine differential expression of transcripts across treatment groups (Love et al. 2014). First, we filtered transcripts that had an average read count of less than one in all samples to reduce noise. The count matrix was then normalized using DESeq2’s default normalization parameters. Differential expression was calculated from the normalized count matrix using the Wald Test for pairwise contrasts of each of our treatment groups (i.e., pathogen vs. control, probiotic vs. control, and pathogen vs. probiotic). The *p* values from this test were corrected using a Benjamini–Hochberg false discovery rate (FDR) method. Transcripts with a p-adjusted value less than 0.1 were considered significantly differentially expressed (10% FDR). An FDR of 0.1 was chosen specifically because of the exploratory nature of this study in identifying associations between larval immunity and bacterial exposure. With that in mind, FDR was not the only metric used to determine whether a gene was significant or biologically relevant. After identifying which transcripts were significantly differentially expressed by FDR, transcripts were further filtered to those with an absolute value of log fold change (LFC) of greater than two to further narrow down transcripts of interest.

### Gene ontology (GO) enrichment

To identify enrichment of broad processes and functions independent of differential expression, we used a Gene Ontology Mann–Whitney U (GO MWU) analysis which performs adaptive clustering of the GO categories assigned to each transcript from reference transcriptome annotations (Voolstra et al. 2011; Wright et al. 2015). The input for this analysis was the LFC output from DESeq2 for all transcripts in the dataset, the annotation file from the reference transcriptome (Changsut et al. 2024), and the gene ontology database file. The output provides a Benjamini–Hochberg FDR-adjusted p value and a delta rank representing the change in average rank of an individual GO term compared to the average rank of all GO terms in the dataset (i.e., magnitude of enrichment).

### Weighted gene correlation network analysis

We used weighted gene correlation network analysis (WGCNA) to cluster transcripts into modules based on similar expression profiles across samples (Langfelder and Horvath 2008). A signed network was constructed using a minimum module size of 50 transcripts and a cut height of 0.25. From there, module eigengenes were correlated with treatments using a Pearson correlation. To run these correlations, we used the binarize function within WGCNA to create a matrix with columns for each pairwise contrast (i.e., pathogen vs. control, probiotic vs. control, and pathogen vs. probiotic). In that matrix, the two groups being compared were represented by one and zero, and the group not in the contrast was represented by NA. We chose to run the contrasts in this manner rather than comparing each group to all other samples to better isolate the effect of each individual treatment relative to control. We then used the correlation outputs to identify modules of interest (i.e., those with strong correlations to treatment groups). Following WGCNA, we used STRINGdb to build functional networks for each module and used a hypergeometric test to calculate enrichment of GO and KEGG (Kyoto Encyclopedia of Genes and Genomes) terms for each module (Szklarczyk et al. 2025). The p value from the hypergeometric test was corrected using the Benjamini–Hochberg FDR method, and a p-adjusted value of less than 0.05 was considered significant enrichment. All analyses and data visualizations were conducted in R version 4.4.3.

## Results

### Larval survival

Larval survival showed clear differences among the treatment groups. All replicates (100 larvae per dish; *n* = 5) within the pathogen treatment group exhibited 100% mortality within 24 h of initial exposure to *V. coralliilyticus* RE22. Prior to death, the pathogen-treated larvae swam in abnormal patterns and developed morphological abnormalities (~ 18–20 h post-exposure). At time of sampling (4 h after exposure), however, pathogen-treated *A. poculata* larvae were still alive and normal in appearance. In contrast, *A. poculata* larvae in the probiont (S4) and control treatments all survived and maintained normal morphology and swimming behavior throughout the experiment. After 24 h, the experiment was concluded and no settlement assays or further monitoring took place.

### Sequencing

Transcriptomic sequencing generated a total of 40,118,656 reads, averaging 2,674,577 reads per sample. Following the cutadapt filtering and deduplicating, 9,513,928 reads were retained, an average of 634,262 reads per sample (24% of the dataset). These reads were then mapped to the reference transcriptome at an average rate of 31.8% mapping per sample (~ 2,949,318 reads mapped). Mapped reads then underwent mean filtering to reduce noise in the dataset, and we retained 2,860,503 reads, an average of 190,700 reads per sample, which were used as input for differential expression analysis.

### Differential expression

**Fig. 1:**
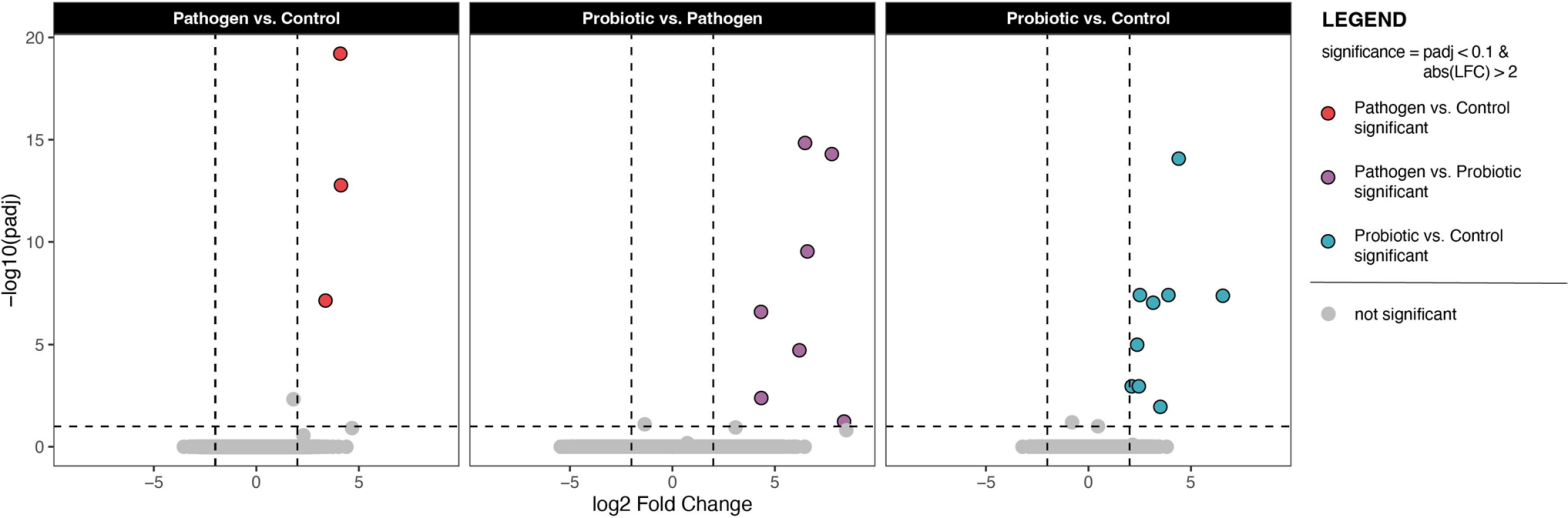
Volcano plot relating the LFC to p-adjusted values on a log scale for each transcript in the dataset in each treatment comparison. Horizontal lines indicate significance thresholds of p.adj = 0.1, while vertical lines indicate ± 2 log2 fold change to indicate a threshold for what we are considering biologically interesting degree of change for further investigation. Gray points are non-significant transcripts, and points in color are considered significant with color indicating the treatment comparison they belong to

**Fig. 2:**
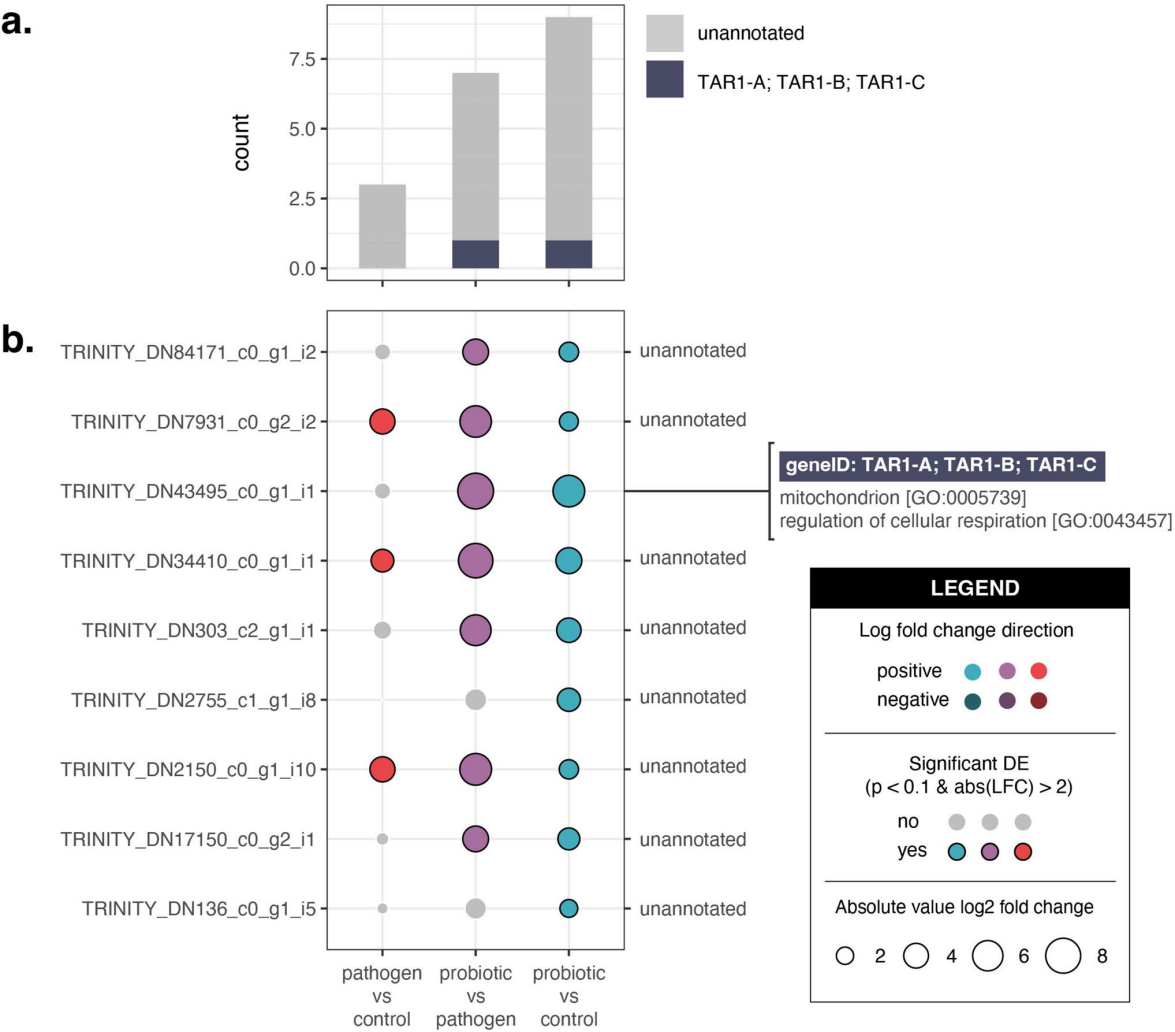
**a** Counts of significantly differentially expressed genes in each treatment comparison, colored based on annotation. **b** Log fold change and annotation information for each of the differentially expressed genes across each treatment comparison

**Fig. 3:**
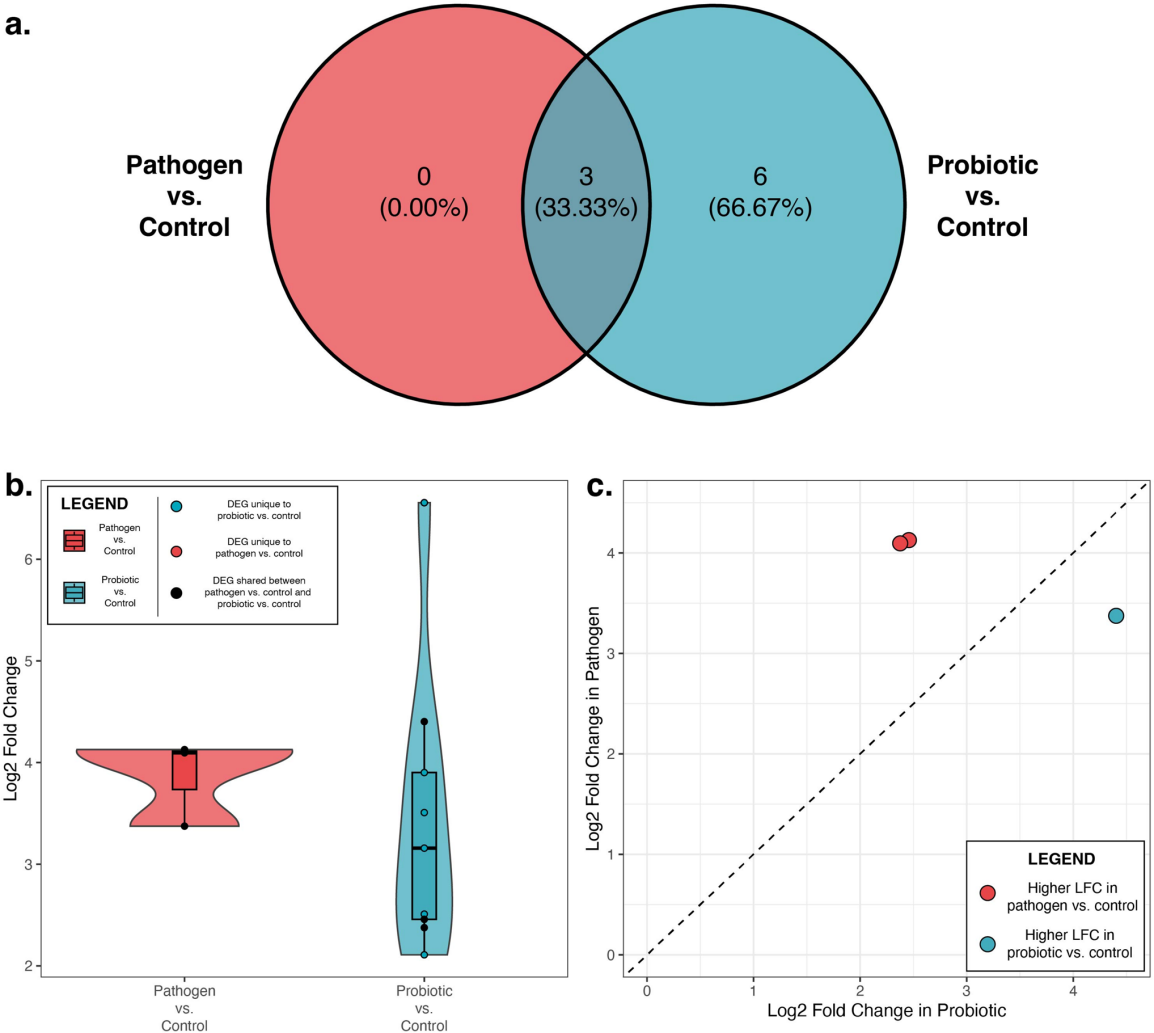
**a** Comparison of differentially expressed genes between the pathogen vs control and probiotic vs control comparisons showing **b** distribution of LFC for differentially expressed transcripts in both comparisons and **c** a comparison of LFC for genes differentially expressed in both comparisons

Both pathogen and probiotic treatments elicited a transcriptional response (compared to control), largely characterized by upregulation of a small set of transcripts (Fig. 1). Transcripts were considered differentially expressed if the absolute value of their LFC was greater than two and if their p-adjusted value was less than 0.1. In the pathogen treatment, three transcripts were differentially expressed compared to control (Fig. 2a), none of which were annotated (Fig. 2b). In the probiotic treatment, nine transcripts were significantly differentially expressed compared to control. Of the significant transcripts, one was annotated as TAR1-A, TAR1-B, TAR1-C, which had GO annotations for mitochondrion (GO:0005739) and regulation of cellular respiration (GO:0043457) (Fig. 2). Seven genes were differentially expressed between the probiotic and pathogen treatments, all of which were upregulated in the probiotic treatment relative to the pathogen (Fig. 2a). The only annotated transcript in this group was again TAR1-A, TAR1-B, TAR1-C, overlapping with the probiotic vs. control comparison (Fig. 2b). Pathogen treatment elicited stronger average LFC changes in a smaller subset of transcripts compared to the probiotic treatment, which induced variable changes in a broader group of transcripts (Fig. 3a, b). Of the genes that were differentially expressed in both comparisons, two were more strongly expressed in the pathogen comparison and one was more strongly expressed in the probiotic comparison (Fig. 3c).

### Gene ontology Mann–Whitney U analysis

**Fig. 4:**
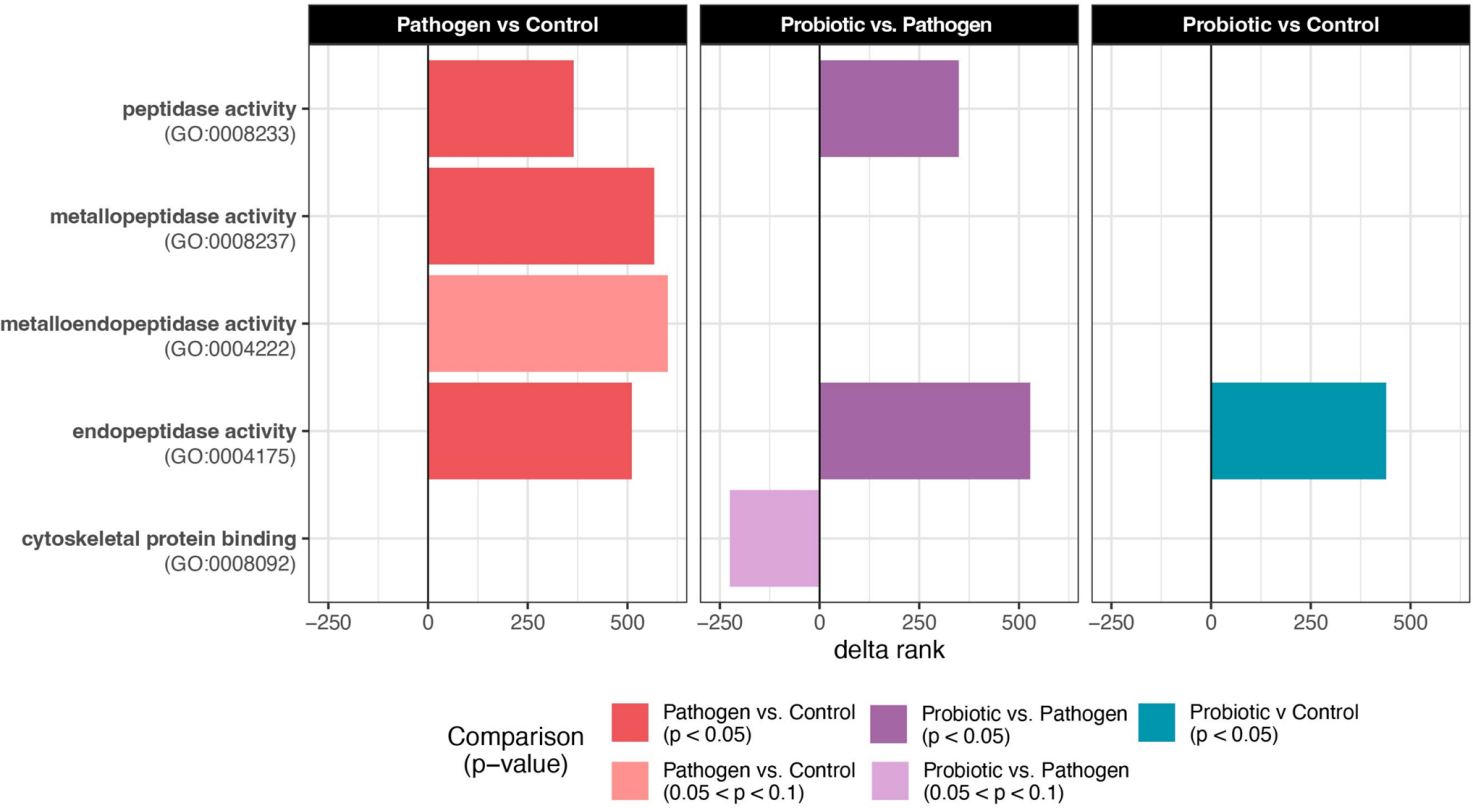
GO MWU results showing the enrichment of significant GO terms in each treatment comparison. Color is used to indicate comparison where the significant terms were enriched. Shade of bar used to indicate *p*-adjusted value (darker shades = *p*-adjusted < 0.05, lighter shades 0.05 < *p*-adjusted < 0.1)

Five GO molecular functions were significantly enriched across at least one of our comparisons. Endopeptidase activity (GO:0004175) was significantly positively enriched (Mann–Whitney U, p.adj < 0.05; Supplemental Table 1) in both pathogen and probiotic treatments, and more strongly enriched in the probiotic treatment compared to the pathogen treatment (Fig. 4). The transcripts contributing to endopeptidase activity in our dataset included those annotated as cathepsins (Ctsb) and presenilins (psen1, Psn). Compared to controls, peptidase activity was significantly activated as a result of pathogen treatment (Mann–Whitney U, p.adj = 0.001), but not as a result of probiont (Mann–Whitney U, p.adj = 0.16, Fig. 4). However, peptidase activity was positively enriched (i.e., higher) in the probiotic treatment, relative to the pathogen treatment (Mann–Whitney U, p.adj = 0.003; Fig. 4). Similar to the endopeptidase group, the transcripts contributing to the peptidase group included those annotated as cathepsins (Ctsb, and cp1), as well as some aminopeptidases (lap-2, Npepl1), NOD-like receptors (nlrp1), and legumain (LGMN). Metallopeptidase activity (GO:0008237; Mann–Whitney U, p.adj = 0.003) and metalloendopeptidase (GO: 0004222; Mann–Whitney U, p.adj = 0.056) were activated in the pathogen treatment, though metalloendopeptidase was only marginally significant (i.e., 0.1 > *padj* > 0.05; Supplemental Table 2; Fig. 4). Transcripts contributing to the metallopeptidase and metalloendopeptidase categories included those annotated as a disintegrin and metalloproteinases (ADAM) and ADAM with thrombospondin motifs (ADAMTS; Adamts7, ADAMTS6, ADAMTS9, ADAM10, ADMATS10, ADAM17), zinc metalloproteinases (ZMPSTE24, dpy-31, BP10, nas-13, nas-15), tolloid-like proteins (tll1, tll2), and matrix metallopeptidases (MMP9, MMP17). Finally, cytoskeletal binding protein (GO:0008092) was more strongly activated in pathogen treatment relative to probiotic treatment (Mann–Whitney U, p.adj = 0.09) (Fig. 4).

### Weighted gene correlation network analysis

**Fig. 5:**
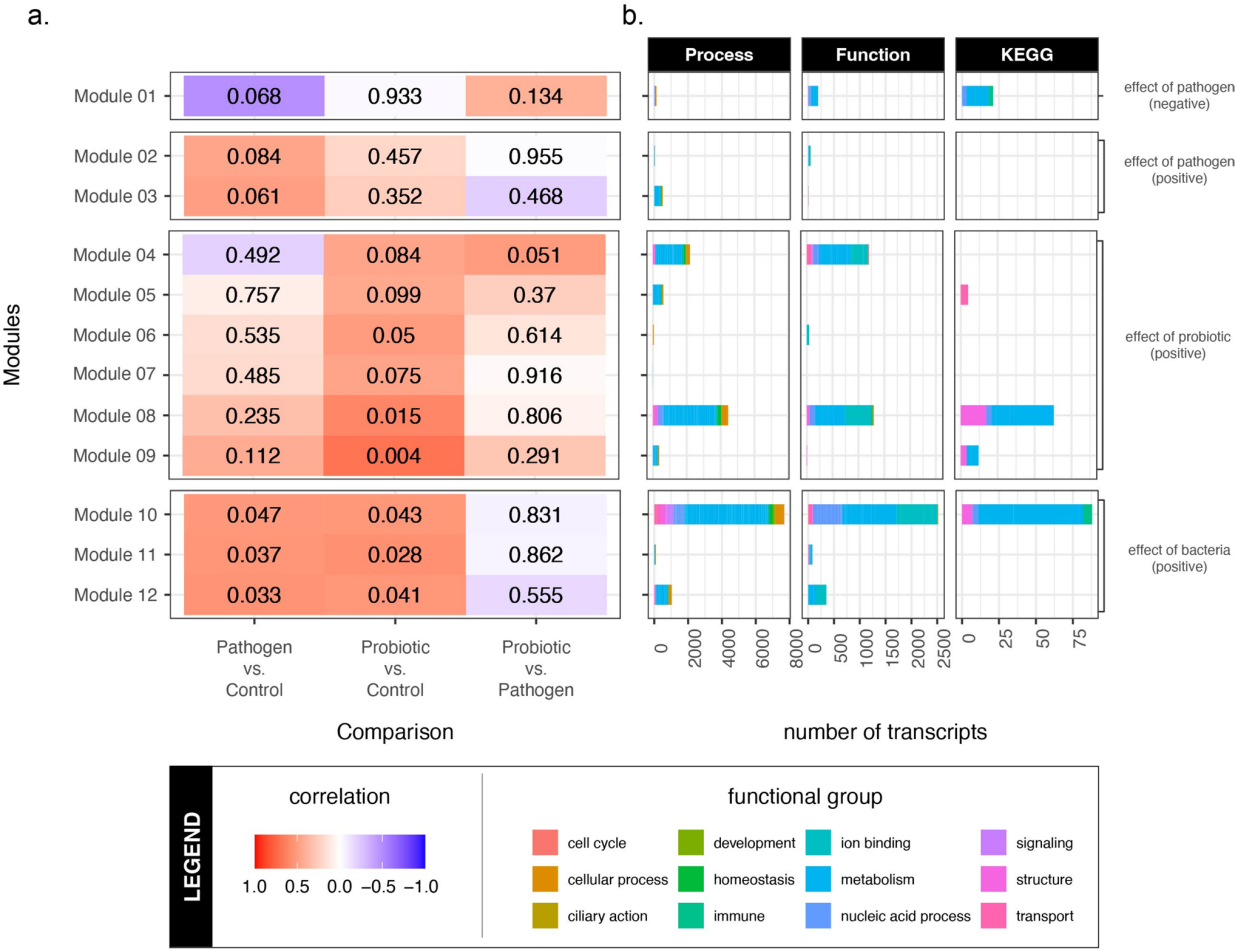
**a** WGCNA correlation between modules and treatment groups with red indicating strong positive correlation, white indicating weak correlation, and blue indicating strong negative correlation. Labels inside each square represent p-adjusted values to indicate significance of correlation. **b** Functional categories of GO (biological process and molecular function) and KEGG processes enriched in each module and the number of transcripts in that module that contributed to each enrichment group. The total bar length does not reflect the total number of transcripts in the module, but rather the number of transcripts in each enriched group contained in that module. Transcripts may be represented multiple times if they contain annotations in multiple functional groups

WGCNA analyses resulted in a network comprised of 36 biologically relevant modules ranging from 79 to 1,731 transcripts and one module containing transcripts that did not map to any module (3,330 transcripts). Of those modules, 12 modules had strong correlations with at least one of the treatment comparisons (i.e., pathogen vs. control, probiotic vs. pathogen, and probiotic vs. control) using a p-adjusted cutoff of 0.1 (Fig. 5a). Of these twelve, three modules were uniquely associated with the pathogen treatment (Modules 01–03), six were uniquely associated with the probiotic treatment (Modules 04–09), and three were associated with both pathogen and probiotic response and therefore were considered a shared response to bacteria (Modules 10–12; Pearson Correlation, p.adj < 0.1; Fig. 5a).

**Fig. 6:**
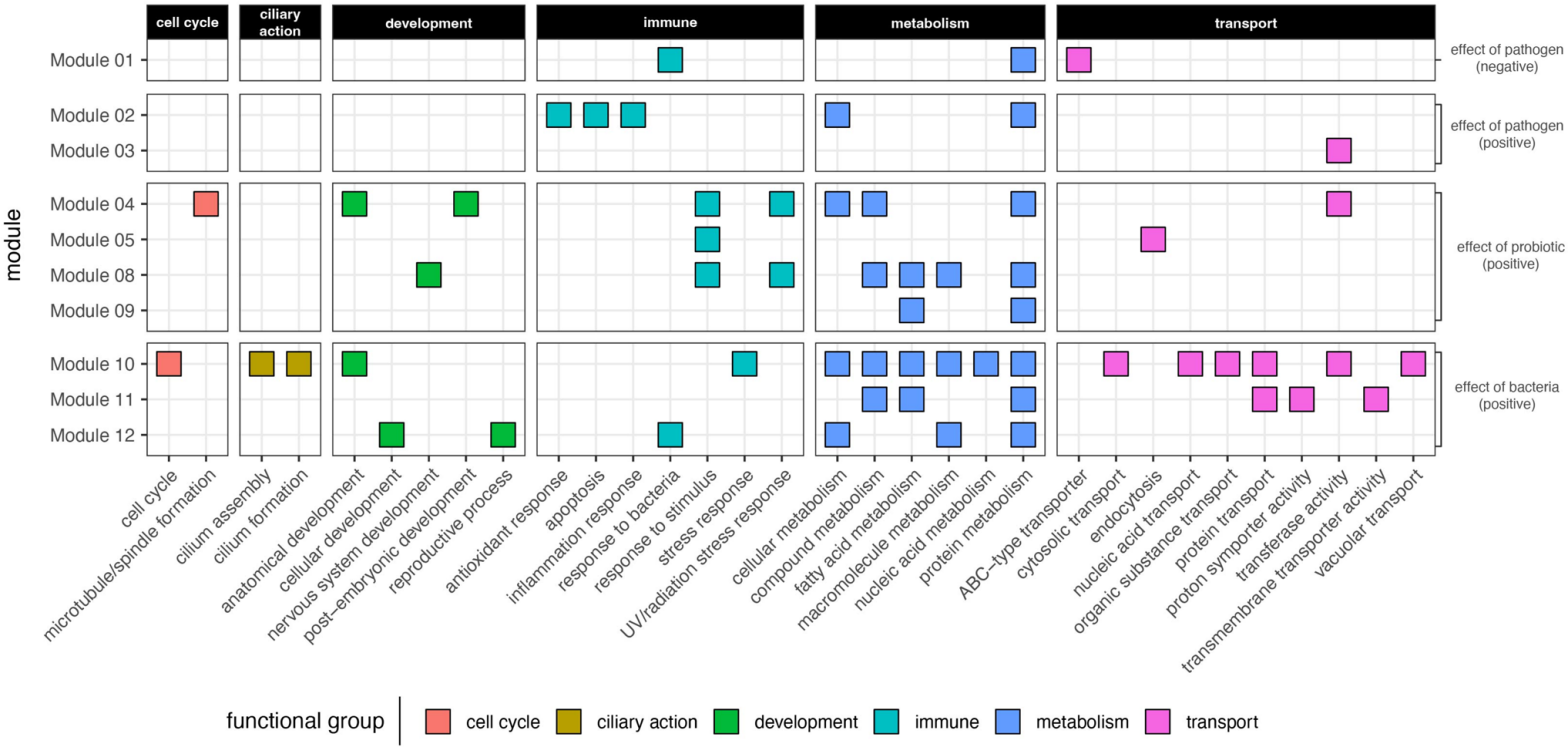
Summary of unique enrichment terms in each module broken down by functional categories. Colors represent functional categories and the presence of a square means at least one of the enriched terms for that module belonged to that group

Enrichment results were filtered to GO biological processes (Process), GO molecular function (Function), and KEGG pathway terms. Each module with the exception of Modules 06 and 07 was enriched for terms in one or more of these three categories. The remaining ten modules were largely enriched for terms related to metabolism, immunity, transport, ion binding, and nucleic acid processes (Fig. 5b). We further filtered to enriched terms that were unique to pathogen response, probiotic response, or shared response to bacteria. Those terms were then collapsed by function to better summarize enriched functions in each module. Terms involved in cell cycle and development processes were enriched in modules that correlate positively with probiotic or shared (pathogenic and probiotic) response to bacteria (hypergeometric test, p.adj < 0.002) (Fig. 6, Supplemental Figures 1–6). Terms associated with ciliary action were only enriched in modules associated with shared response to bacteria (hypergeometric test, p.adj < 0.002; Fig. 6, Supplemental Figures 1–6). Immune terms showed distinct enrichment between probiotic- and pathogen-associated modules. Pathogen-associated modules were enriched for apoptosis, inflammation response, and antioxidant activity (hypergeometric test, p.adj < 0.05), while probiotic associate modules were enriched for response to radiation and response to stimuli (hypergeometric test, p.adj < 0.001; Fig. 6, Supplemental Figures 1–6). Metabolic terms were the most abundant group of terms enriched across our modules. Fatty acid metabolism and more generally macromolecule metabolism terms were enriched only in modules associated with either the shared response to bacteria or the response to probiotic (hypergeometric test, p.adj < 0.01; Fig. 6, Supplemental Figures 1–6). Finally, several terms associated with cellular transport were enriched in the modules representing shared response to bacteria (hypergeometric test, p.adj < 0.01). However, endocytosis was unique to a probiotic response module (hypergeometric test, p.adj = 0.025), while ABC-type transporter was unique to a pathogen response module (hypergeometric test, p.adj = 0.03; Fig. 6, Supplemental Figures 1–6).

## Discussion

Here, we present an experimental approach investigating the roles of *A. poculata* larval immunity in response to diverse bacterial stimuli. Our results reveal shared host responses to probiont and pathogen, as well as signatures specific to each the pathogen and probiont treatments. While there was considerable overlap in pathogen and probiont response, pathogen treatment elicited stronger transcriptional responses (higher average LFC), and probiont treatment yielded a broader response (more unique differentially expressed genes). The functional and sequence clustering analyses revealed distinct cellular mechanisms associated with each response.

### The role of peptidases in bacterial response

Functional enrichment analyses revealed significant changes in various peptidase activity processes as a result of exposure to bacteria (compared to controls), including metallopeptidase activity (GO:0008237), metalloendopeptidase activity (GO:0004222), endopeptidase activity (GO:0004175), and peptidase activity (GO:0008233). Peptidases are enzymes responsible for breaking peptide bonds in proteins, which can play a wide range of roles in protein breakdown, protein modification, immune responses, and cellular signaling (Page and Di Cera 2008). Several classes of peptidases with diverse functions exist, including metallopeptidases, peptidases that require metal ions to cleave the peptide bond, and endopeptidases, peptidases that cleave peptide bonds in the middle of the molecule as opposed to the ends. Notably, we observe differentiation in peptidase activation across stimuli. The more general term endopeptidase activity was characteristic of general response to bacteria (i.e., enriched in response to both pathogen and probiotic compared to control), whereas metallopeptidase activity and metalloendopeptidase activity were specific to pathogen response alone.

Peptidases can serve a wide range of roles in coral immunity, including cell growth and remodeling, extracellular matrix (ECM) remodeling, and inflammation response (Libro et al. 2013; Takagi et al. 2020). In our dataset, endopeptidase activity, which was activated in response to both bacterial treatments, was the only biological process enriched in the probiont treatment. In both probiont and pathogen treatments, endopeptidase activity enrichment included expression of transcripts related to developmental processes and inflammation responses. These transcripts include ones for presenilin (PSEN1, Psn), which is part of the γ-secretase complex and is involved in the NOTCH signaling pathway (Zhang et al. 2013), an essential pathway in endoderm development and differentiation of immune cells during development (Brunkan and Goate 2005). This finding suggests that immune cell development is affected by bacterial exposure, regardless of nature (probiotic or pathogenic). Likewise, there was increased expression of cathepsins (Ctsb) and ADAM metalloproteinases, which have previously been shown to direct the inflammation response, again pointing to a shared immune response against general bacterial stimuli (Yadati et al. 2020; Primakoff and Myles 2000). Combined, our results identify peptidases as a main component of the host’s early life stage response to bacterial exposure, and of its developing immune system. These results provide novel insights on how marine invertebrates may respond to bacterial exposures despite the lack of mature allorecognition systems documented in studies of other marine invertebrates (Puill-Stephan et al. 2012).

The unique enrichment of metallopeptidase activity and metalloendopeptidase activity in the response to the pathogen highlight a capacity for specialization in larval immune responses. One-third of the transcripts driving this enrichment belonged to either the ADAM family, the ADAMTS family, or the astacin family (zinc metalloproteinases and tolloid-like proteins), which specifically play roles in regulation of innate immune response via mechanisms like NF-κB signaling, inflammation response and wound healing (Soria-Valles et al. 2016; Levy and Mass 2022; Caley et al. 2015; Khokha et al. 2013). The ADAM and ADAMTS genes appear especially important to the enrichment results given their prevalence in our dataset. The ADAM metallopeptidases are transmembrane peptidases functioning in adhesion, protein breakdown, and inflammation (Primakoff and Myles 2000). The ADAMTS metallopeptidases serve similar functions, but contain an additional thrombosponin-1 like repeat, and are located in the extracellular matrix (ECM). In addition to the functions they share with ADAM, they also play a role in ECM structuring and transforming growth factor-β (TGF-β) signaling (Tang 2001; Cain et al. 2022). For example, ADAMTS6, which contributes to observed patterns, has known functions in microfibril formation and turnover which can affect other molecules’ ability to move through the ECM (Mead 2022) and therefore may impact the pathogen’s ability to reach the host cell. Notably, these patterns are unique to pathogen treatment, suggesting that alteration of ECM composition is a potential mechanism for pathogen defense in larvae. Another gene in the dataset contributing to metallopeptidase enrichment is ADAMTS10, which is closely related to ADAMTS6. These genes are often linked in positive feedback loops and play roles in activation of TGF-β (Mead 2022; Tang 2001; Cain et al. 2022). TGF-β is a known regulator of cnidarian immunity (Fuess et al. 2020), and thus, enrichment of these transcripts in response to pathogen stimuli may point to a broader role of TGF-β immune regulation in larval pathogen defense. Notably, TGF-β also has putative roles in regulation of beneficial symbiosis with algae (Detournay et al. 2012), but these transcripts were not differentially regulated in response to the probiont in this study. Consequently, our results point to important nuances, molecular cross talk, and specialization in immune regulation that may direct early life stage response to beneficial and antagonistic partners.

### Unique and shared defense mechanisms revealed by coexpression analyses

Research in other cnidarian systems has identified the importance of physical defense mechanisms and innate immune components in response to both probiont and pathogen exposures. Ciliary action is a physical defense mechanism well-documented in adult tropical corals as a first line of defense (Gavish et al. 2021; Bakshani et al. 2018) and in *A. poculata* (Changsut et al. 2024). Our network analysis identifies numerous transcripts involved in ciliary action in the host response to both pathogen and probiont, indicating that these responses are general to bacterial stimulus regardless of the nature (i.e., pathogenic or beneficial). These same modules associated with general response to bacteria were also enriched for the immune terms “Defense response to gram-negative bacteria” and “Response to stress.” Many of the transcripts contributing to these terms in our dataset were involved in LPS binding, TLR signaling, and regulation of NFκ-B signaling, all of which have been identified in other cnidarian systems as important in the response to beneficial and harmful microbes and also in the regulation and management of the host microbiome (Bosch 2013; Gao et al. 2021). These same responses were also identified as conserved responses to bacterial stimuli in a study exposing adult corals to probiotic and pathogenic bacteria (Van De Water et al. 2018), further highlighting similarities in immune responses across life stages.

Network analyses further revealed unique transcriptomic associations in pathogen-exposed larvae that distinguished the response to pathogen from the response to other bacterial stimuli. The pathogen response included modules enriched for apoptotic processes (“Apoptotic DNA fragmentation”), inflammation response (“Metalloendopeptidase inhibitor activity”), and antioxidant response (“Catalase activity” and “Hydrogen peroxide catabolic process”). Notably, terms associated with pathogen response have been characterized in previous studies of cnidarian pathogen response (Traylor-Knowles and Connelly 2017; Mydlarz et al. 2016). Apoptosis, programmed cell death, has documented roles in regulation of symbiosis in adult corals during times of stress (environmental and disease), in pathogen defense, and in the regulation of morphogenesis and development in invertebrates (Pernice et al. 2011; Fuess et al. 2017; Dunn and Weis 2009; McFall-Ngai and Ruby 1991; Nyholm and McFall-Ngai 2004). Antioxidant activity in cnidarians occurs in response to reactive oxygen species which can come from heat and other environmental stressors, the algal symbiont in adult corals, or can be produced by bacterial pathogens during infection (Weis 2008; Hawkins et al. 2015; Palmer et al. 2011). Given that the role metallopeptidases can play in regulating the timing and amplitude of signals responsible for antioxidant, inflammation, apoptotic responses, and more in other biological systems (Khokha et al. 2013), our GO enrichment results provide further context for the host immune response and its role in defense.

In contrast, processes associated with response to probiotic bacteria are similar to those previously documented in adult corals as responses to abiotic environmental stressors (Traylor-Knowles and Connelly 2017). The enriched immune terms for modules associated only with response to probiont included “Response to stimulus,” “Response to UV,” and “Response to radiation.” These terms may reflect a detrimental effect of the probiont on the larvae by triggering stress responses, but could also demonstrate activation of pathways early in development that convey protection from those stressors in the future. Enrichment of response to UV radiation among the probiotic-exposed larvae is of particular interest, as probiotic treatments are often used in adult corals to develop resilience to these stressors. Stress caused by UV radiation can lead to effects including decreased skeleton deposition and growth rates, oxidative stress from reactive oxygen species, bleaching, mortality, and more (Shick et al. 1996). A number of studies have documented that the introduction of beneficial microbial consortia (BMC) to corals can successfully mitigate these effects on both the adult coral and its algal symbiont (De Breuyn et al. 2025; Ashraf et al. 2024; Moradi et al. 2023). Such benefits may also be common even in aposymbiotic early life stages though there are limited data on the molecular pathways commonly activated in response to BMCs. *Phaeobacter inhibens* S4, the probiont used in this study, has previously been shown to activate a wide range of immune signaling pathways and immune effectors in *Crassostrea virginica* (eastern oyster) larvae, allowing them to develop a defense response against pathogens in as little as 24 h prior to infection (Modak and Gomez-Chiarri 2020). In this study, the enriched terms in response to the probiont *P. inhibens* S4 also contain transcripts for a number of PRRs suggesting potential similar effects of the probiont on coral and oyster larvae. In summary, the unique responses observed following probiotic stimuli include the activation of beneficial abiotic stress response pathways, consistent with patterns observed in adult corals. Identification of these pathways provides an opportunity for further work focused on the underlying mechanisms of probiotic-mediated coral resilience for restoration and recovery initiatives.

### Potential benefits of probionts on development and metabolism

Patterns of enrichment across coexpression modules revealed additional signatures pointing to potential nutritional impacts on larval development. Developmental and fatty acid and macromolecule metabolic processes were enriched in modules associated with probiont and general bacterial response. Enrichment of fatty acid metabolism in probiont-associated modules is consistent with recent findings that probionts have the potential to enhance growth of adult corals through supplementing their nutrition (Raimundo et al. 2024; Peixoto et al. 2021; Doering, et al. 2023). We hypothesize that similar mechanisms may be especially beneficial for small planktotrophic (feeding) larvae such as *A. poculata*. In marine planktotrophic larvae, fatty acid nutrition, acquired primarily through feeding, is essential to healthy larval growth and development (Da Costa et al. 2015). In culture conditions, probiotic bacteria may serve as an important fatty acid nutritional source, which would be particularly beneficial for *A. poculata* larvae that are relatively small (~ 100 µm length) and therefore are limited in what food they can consume. Future work using metabolomics to characterize and quantify fatty acids in *A. poculata* larvae in feeding assays and with probiotics exposure will help to further elucidate these mechanisms.

Beyond nutrition and growth, there were a number of modules related to host development enriched in the probiont treatment. Specific microbes associated with the surface of certain crustose coralline algae (CCA) species induce settlement and metamorphosis in coral larvae (Thompson et al. 2015; Negri et al. 2001; Morse et al. 1988; Sneed et al. 2015, 2014). The probiont-associated transcripts involved in morphogenesis, anatomical development, and cellular development with probiotic exposure in this study further support the capacity for additional bacteria to modulate *A. poculata* development. Further work on the host immune response to species-specific bacterial exposure during early life stages may reveal cellular processes significant to morphogenesis and metamorphosis, and the onset and maintenance of specific microbes/microbiome composition in coral early life stages. Specific regulation of beneficial microbiome assembly during early life stages in corals is critical, particularly because most coral species appear to be colonized by bacteria and archaea from the water column either during planula stages, or after settlement (Sharp et al. 2010, 2012; Ceh et al. 2012; Apprill et al. 2009). Using probiotic treatments to augment development, growth, and microbiome composition of early life stages of corals represents an intriguing opportunity to boost survival in these highly vulnerable stages—both in land-based culture systems and in benthic ecosystems subject to warming seawater and altered microbial landscapes (Apprill and Salerno 2025). Though assessing the effects of the probiont on settlement and colony growth early in development was beyond the scope of this study, the data presented here suggest that as a compelling future direction for this work.

Finally, the absence of unique developmental and metabolism terms in pathogen response modules may be indicative of a negative or neutral effect of pathogen exposure on larval development and growth. While the probiont used in this study may provide supplemental nutrition to larvae, aiding in growth and development, exposure to pathogenic bacteria may have the opposite effect. Induction of defense responses may draw energetic resources away from development and growth, hampering larval success. As coral larvae have limited time to reach developmental milestones before settling (K. Miller and Mundy 2003; J. R. Wilson and Harrison 1998), such effects may significantly impact settlement success rates (Freire et al. 2019). Overall, our enrichment results highlight disparate effects of pathogenic and probiotic bacterial exposure on larval development, which are largely consistent with those observed in adult corals. Taken together, these results point to specific processes that some microbes may provide benefit to the success and survival of coral early life stages.

### Conclusions

Here, we use laboratory manipulations of larvae from the temperate coral *A. poculata* to identify unique and shared responses to pathogenic and probiotic bacteria to better understand the impacts of larvae-bacteria interactions in early life stages of the host. Our dataset suggests that immune-related peptidases are involved in recognizing and responding to bacterial stimuli in general. Yet, pathogenic and probiotic bacteria also elicit unique responses, and we hypothesize that these responses could have substantial implications on energy allocated for holobiont survivorship and growth. Pathogen responses highlight a role for metallopeptidases in defense response, specifically in activation of inflammation and TGF-β to regulate the immune response. Further association of transcripts involved in apoptosis, antioxidant, and inflammation with pathogen response suggests larvae are capable of a diverse immune response to pathogens characterized by pathways involved in adult immunity. In contrast, probiotic exposure was associated with potentially beneficial activation of pathways related to abiotic stress response, nutrition, growth, and development. Collectively, these suggest a potential benefit of probiotic exposure to larvae with positive impacts on energetic budget and development. A mechanistic approach is required to fully resolve the implications of bacterial exposure on the immune response and survival of early life stages of corals. The data in this study demonstrate the importance of the immune system in response to bacteria during early life stages in corals, characterize important functions within the larval immune system in response to unique stimuli, and highlight potential metabolic and developmental benefits of exposing coral to probiotics during larval development. Our data provide a foundation for more experimentation aimed at elucidating underlying mechanisms that drive coral larval responses to pathogens and to probiotics, and for informing applications for enhancement of larval and post-settlement survival of corals—both those grown in laboratory-based restoration efforts, and those in altered wild reef ecosystems, as coral disease continues to increase in prevalence across tropical reefs across the globe.

## Supplementary Information

Below is the link to the electronic supplementary material.

Supplementary file1 (DOCX 925 kb)

## Data Availability

All sequences are uploaded to NCBI BioProject PRJNA1266695. All metadata and scripts are publicly available in https://github.com/ErinBorbee/AstrangiaLarval_bacteriaExposure.
