# Supplementary material for: Coral larvae have unique transcriptomic responses to pathogenic and probiotic bacteria
Source: Coral Reefs. 2026 Feb 24;45(3):1119–34. doi: 10.1007/s00338-026-02830-1 (PMC13282300; doi:10.1007/s00338-026-02830-1)
Supplement: Supplementary file 1 — Supplementary file1 (DOCX 925 kb) [file 338_2026_2830_MOESM1_ESM.docx]

**Supplementary material for:**

**Coral larvae have unique transcriptomic responses to pathogenic and probiotic bacteria**

Erin M. Borbee^1^, Isabella V. Changsut^1^, Kira Bernabe^2^, Alicia Schickle^3^, David Nelson^2*^, Koty H. Sharp^3^, and Lauren E. Fuess^1^

^1^Texas State University, Department of Biology, San Marcos, TX USA 78666

^2^University of Rhode Island, Department of Biological Sciences, Kingston, RI USA 02886

^3^Roger Williams University, Department of Biology, Marine Biology, and Environmental Sciences, Bristol, RI USA 02809

*Author was not able to provide feedback on final manuscript due to health reasons

**Supplemental figures**

**
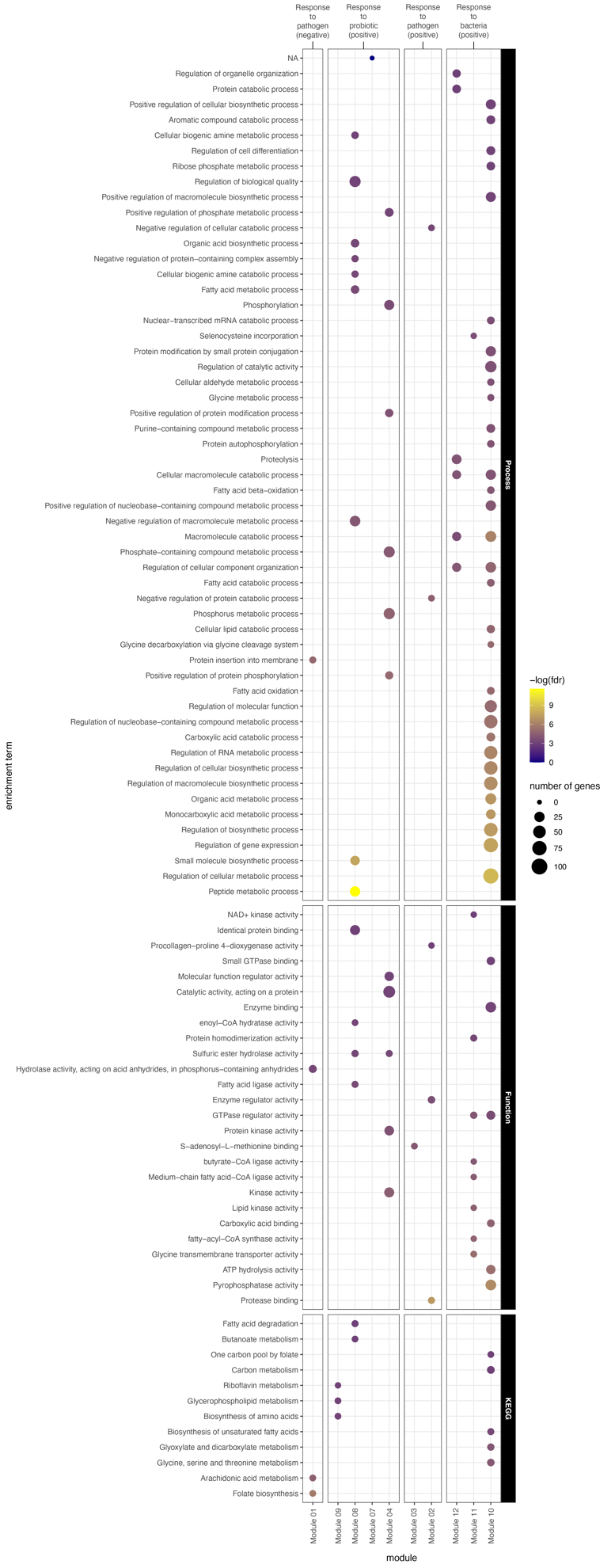
**

**Supplemental Fig. 1** Unique enrichment results for metabolic GO and KEGG terms for each module. GO categories represented include molecular function (Function) and biological process (Process). The x-axis indicates the module with enriched terms, and the y-axis shows the enriched terms. Size of the points represents the number of genes contributing to that term in each module, and the color represents the significance of that term on a log scale (yellow = more significant, blue = less significant). All terms present had a maximum p-value of 0.05

**
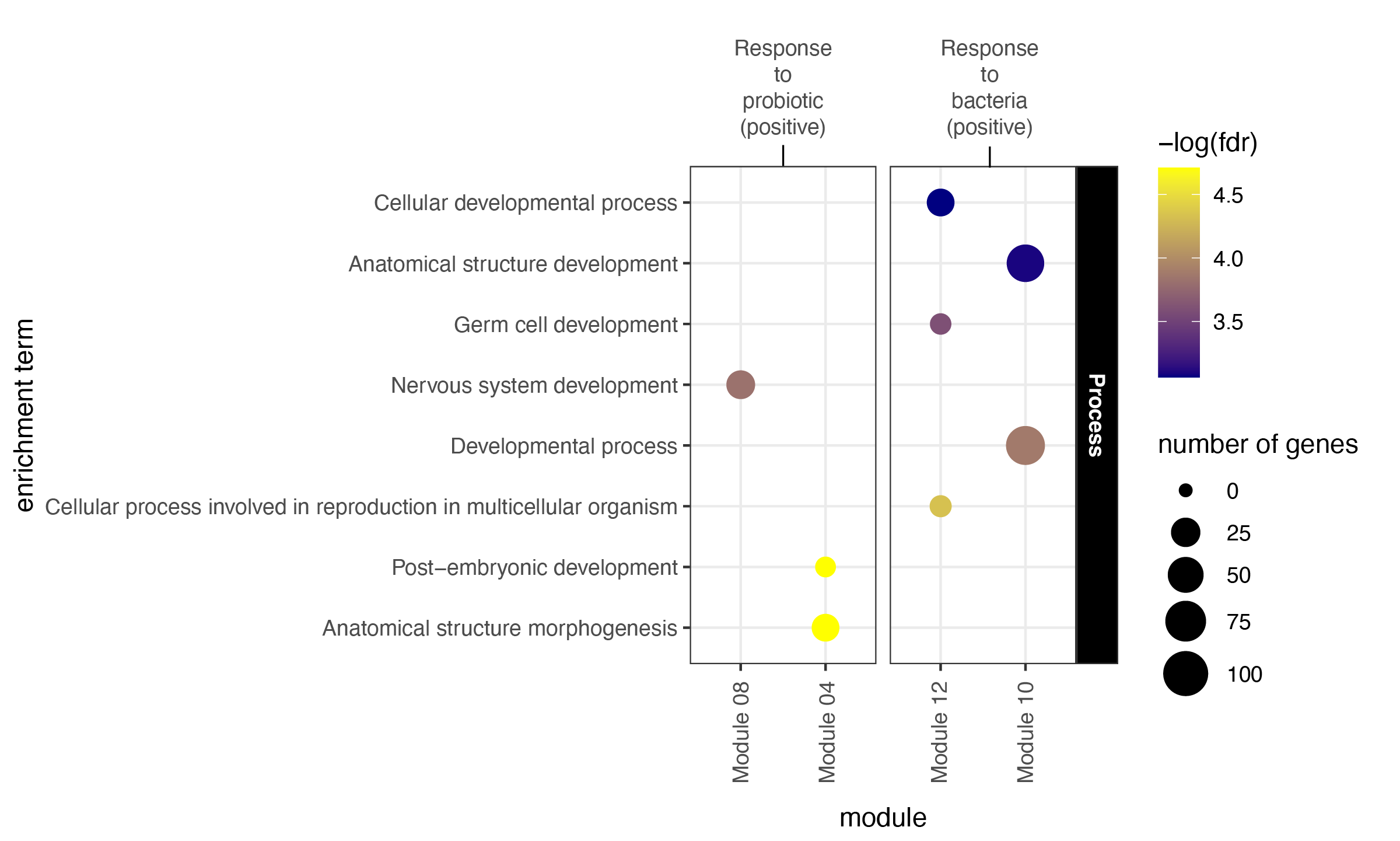
**

**Supplemental Fig. 2** Unique enrichment results for development GO and KEGG terms for each module. GO categories represented include molecular function (Function) and biological process (Process). The x-axis indicates the module with enriched terms, and the y-axis shows the enriched terms. Size of the points represents the number of genes contributing to that term in each module, and the color represents the significance of that term on a log scale (yellow = more significant, blue = less significant). All terms present had a maximum p-value of 0.05


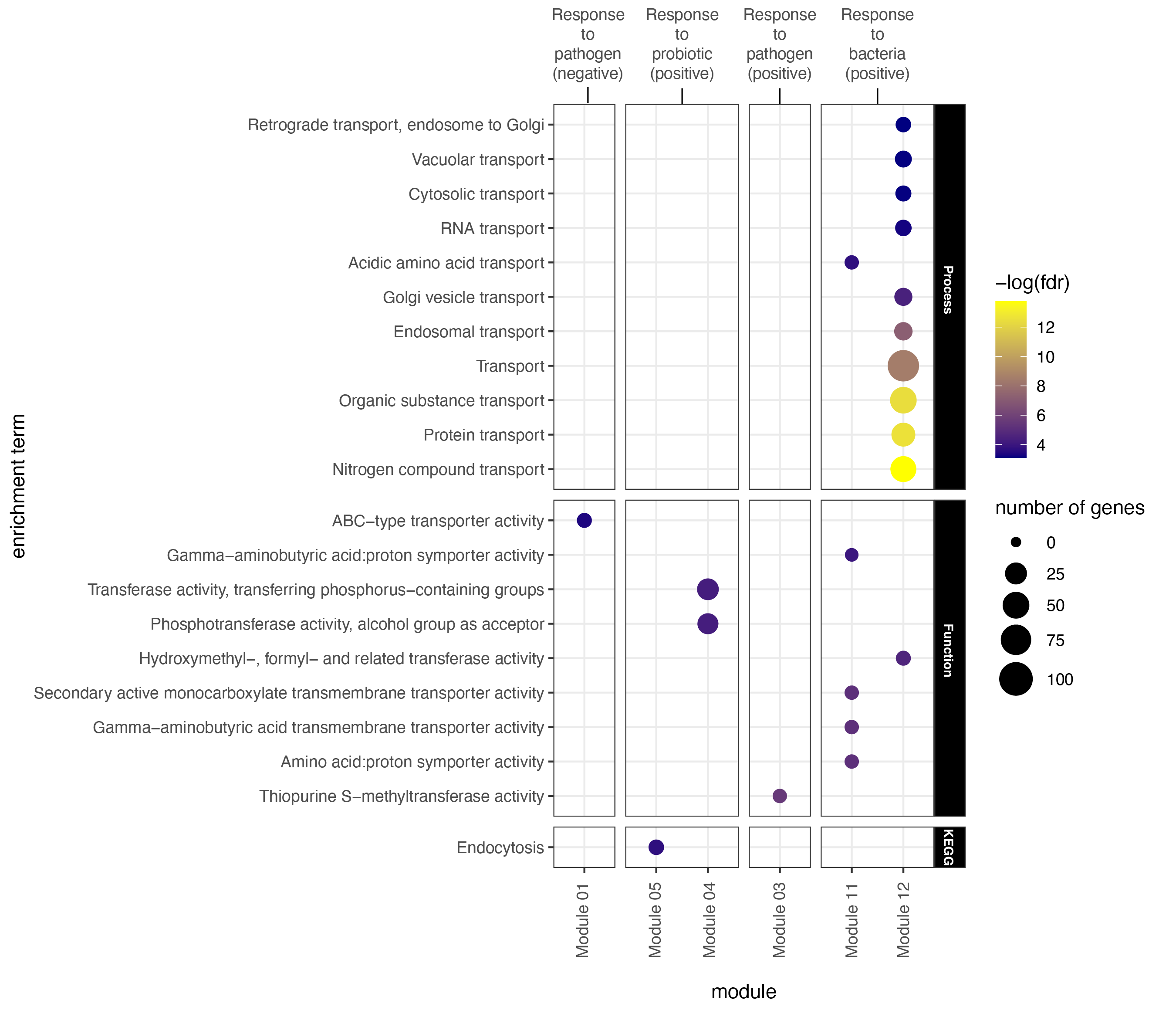


**Supplemental Fig. 3** Unique enrichment results for transport GO and KEGG terms for each module. GO categories represented include molecular function (Function) and biological process (Process). The x-axis indicates the module with enriched terms, and the y-axis shows the enriched terms. Size of the points represents the number of genes contributing to that term in each module, and the color represents the significance of that term on a log scale (yellow = more significant, blue = less significant). All terms present had a maximum p-value of 0.05

**
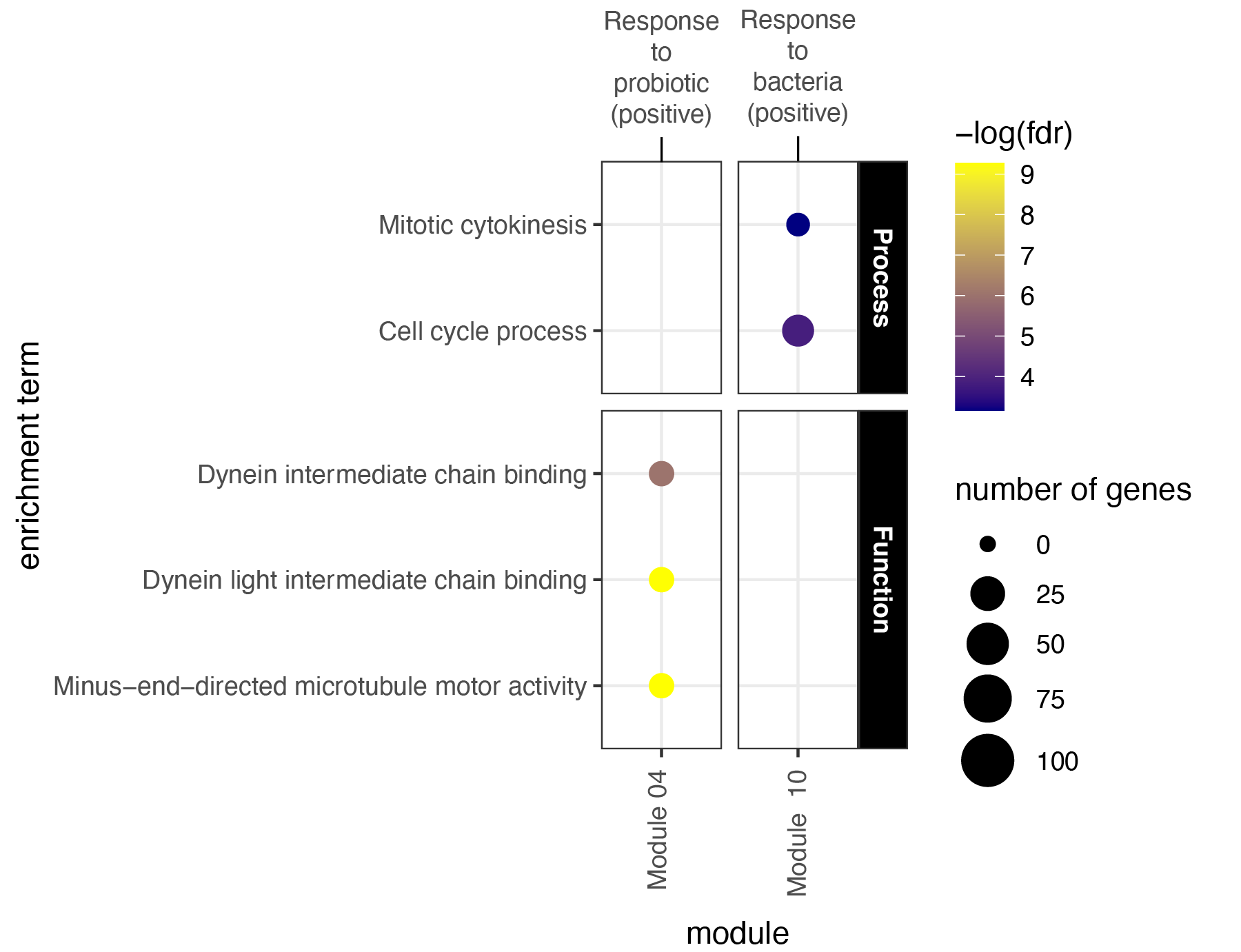
**

**Supplemental Fig. 4** Unique enrichment results for cell cycle GO and KEGG terms for each module. GO categories represented include molecular function (Function) and biological process (Process). The x-axis indicates the module with enriched terms, and the y-axis shows the enriched terms. Size of the points represents the number of genes contributing to that term in each module, and the color represents the significance of that term on a log scale (yellow = more significant, blue = less significant). All terms present had a maximum p-value of 0.05

**
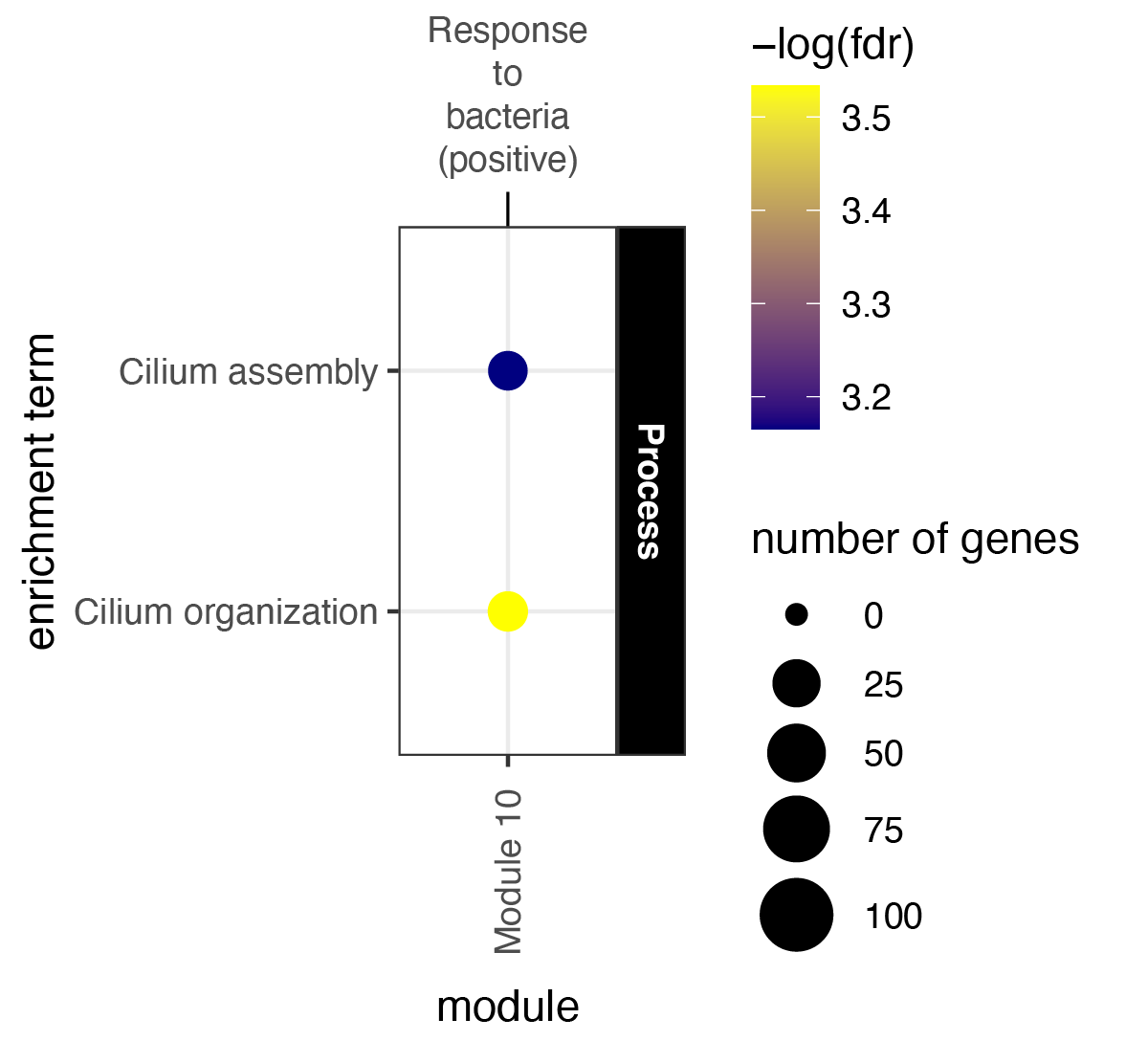
**

**Supplemental Fig. 5** Unique enrichment results for ciliary action GO and KEGG terms for each module. GO categories represented include molecular function (Function) and biological process (Process). The x-axis indicates the module with enriched terms, and the y-axis shows the enriched terms. Size of the points represents the number of genes contributing to that term in each module, and the color represents the significance of that term on a log scale (yellow = more significant, blue = less significant). All terms present had a maximum p-value of 0.05


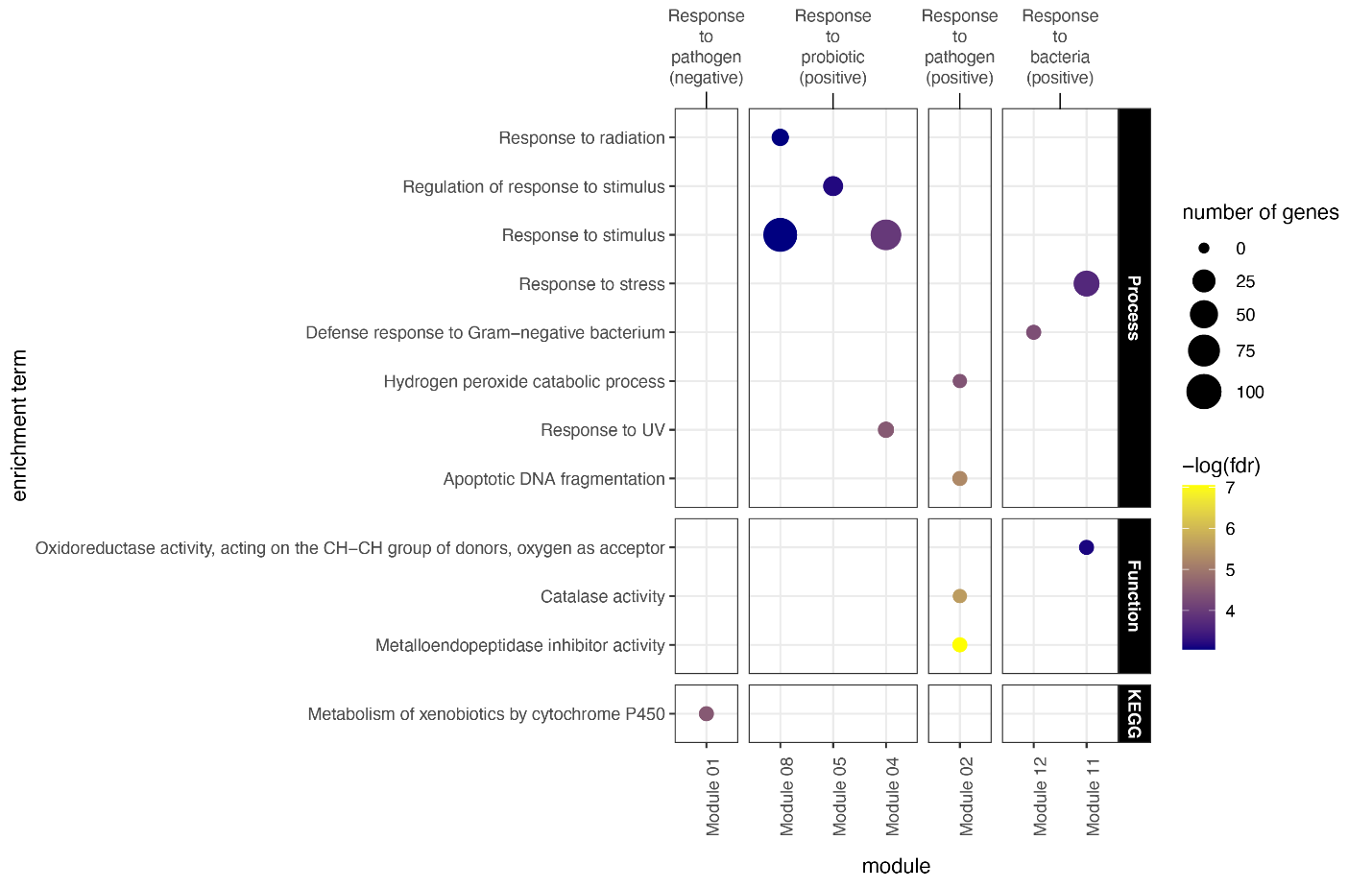


**Supplemental Fig. 6** Unique enrichment results for immune GO and KEGG terms for each module. GO categories represented include molecular function (Function) and biological process (Process). The x-axis indicates the module with enriched terms, and the y-axis shows the enriched terms. Size of the points represents the number of genes contributing to that term in each module, and the color represents the significance of that term on a log scale (yellow = more significant, blue = less significant). All terms present had a maximum p-value of 0.05

**Supplemental Tables**

**Supplemental Table 1.** Significant differential expression output from DESeq2 where LFC is log2 fold change and p.adj represents the p-value corrected using the Benjamini-Hochberg adjustment for transcripts where p.adj < 0.1.

| TranscriptID | baseMean | LFC | lfcSE | stat | pvalue | padj | comparison |
| --- | --- | --- | --- | --- | --- | --- | --- |
| TRINITY_DN7931_c0_g2_i2 | 213.447901 | 4.09527378 | 0.40356341 | 10.1477828 | 3.39E-24 | 6.21E-20 | Pathogen vs Control |
| TRINITY_DN2150_c0_g1_i10 | 58.8142163 | 4.12732419 | 0.48538702 | 8.50316143 | 1.84E-17 | 1.69E-13 | Pathogen vs Control |
| TRINITY_DN34410_c0_g1_i1 | 162.008139 | 3.37413964 | 0.49725536 | 6.78552699 | 1.16E-11 | 7.07E-08 | Pathogen vs Control |
| TRINITY_DN84171_c0_g1_i2 | 37.7043157 | 1.81238988 | 0.37097067 | 4.88553423 | 1.03E-06 | 0.00472576 | Pathogen vsControl |
| TRINITY_DN34410_c0_g1_i1 | 162.008139 | 4.40288173 | 0.49638733 | 8.86985111 | 7.32E-19 | 8.48E-15 | Probiotic vs Control |
| TRINITY_DN303_c2_g1_i1 | 18.0173743 | 3.90160058 | 0.57193989 | 6.82169687 | 9.00E-12 | 3.81E-08 | Probiotic vs Control |
| TRINITY_DN84171_c0_g1_i2 | 37.7043157 | 2.50908882 | 0.36852379 | 6.80848533 | 9.86E-12 | 3.81E-08 | Probiotic vs Control |
| TRINITY_DN43495_c0_g1_i1 | 11.998099 | 6.5561232 | 0.97048977 | 6.75547896 | 1.42E-11 | 4.12E-08 | Probiotic vs Control |
| TRINITY_DN17150_c0_g2_i1 | 14.2545638 | 3.15794288 | 0.47789892 | 6.60797243 | 3.90E-11 | 9.02E-08 | Probiotic vs Control |
| TRINITY_DN7931_c0_g2_i2 | 213.447901 | 2.37585905 | 0.40697513 | 5.83784819 | 5.29E-09 | 1.02E-05 | Probiotic vs Control |
| TRINITY_DN136_c0_g1_i5 | 20.1209165 | 2.10864985 | 0.42648046 | 4.94430587 | 7.64E-07 | 0.00110564 | Probiotic vs Control |
| TRINITY_DN2150_c0_g1_i10 | 58.8142163 | 2.45755296 | 0.49515324 | 4.96321698 | 6.93E-07 | 0.00110564 | Probiotic vs Control |
| TRINITY_DN2755_c1_g1_i8 | 3.82180518 | 3.50786925 | 0.78851464 | 4.4487053 | 8.64E-06 | 0.01111064 | Probiotic vs Control |
| TRINITY_DN9745_c0_g1_i1 | 50.6357192 | -0.7982851 | 0.19793949 | -4.0329756 | 5.51E-05 | 0.06374932 | Probiotic vs Control |
| TRINITY_DN1781_c2_g1_i10 | 131.6918 | 0.46458522 | 0.11899742 | 3.90416216 | 9.46E-05 | 0.09949488 | Probiotic vs Control |
| TRINITY_DN7931_c0_g2_i2 | 213.447901 | 6.47113282 | 0.70998502 | 9.1144639 | 7.91E-20 | 1.45E-15 | Probiotic vs Pathogen |
| TRINITY_DN34410_c0_g1_i1 | 162.008139 | 7.77702136 | 0.8736778 | 8.90147531 | 5.51E-19 | 5.05E-15 | Probiotic vs Pathogen |
| TRINITY_DN2150_c0_g1_i10 | 58.8142163 | 6.58487716 | 0.87367617 | 7.53697692 | 4.81E-14 | 2.94E-10 | Probiotic vs Pathogen |
| TRINITY_DN84171_c0_g1_i2 | 37.7043157 | 4.3214787 | 0.65915285 | 6.55611016 | 5.52E-11 | 2.53E-07 | Probiotic vs Pathogen |
| TRINITY_DN303_c2_g1_i1 | 18.0173743 | 6.19689766 | 1.06110145 | 5.84006146 | 5.22E-09 | 1.91E-05 | Probiotic vs Pathogen |
| TRINITY_DN17150_c0_g2_i1 | 14.2545638 | 4.33454852 | 0.8971184 | 4.83163484 | 1.35E-06 | 0.00413607 | Probiotic vs Pathogen |
| TRINITY_DN43495_c0_g1_i1 | 11.998099 | 8.37466804 | 1.97464455 | 4.24110155 | 2.22E-05 | 0.05823099 | Probiotic vs Pathogen |
| TRINITY_DN9745_c0_g1_i1 | 50.6357192 | -1.3502214 | 0.32618407 | -4.1394463 | 3.48E-05 | 0.07975132 | Probiotic vs Pathogen |

**Supplemental Table 2.** GO MWU results for significant GO terms in each comparsion. Delta rank represents the difference between the average Mann-Whitney rank for that GO term and the average rank of all GO terms in the dataset and p.adj represents the p-value adjusted for false discovery using the Benjamini-Hochberg method.

| delta.rank | pval | level | nseqs | term | name | p.adj | comparison |
| --- | --- | --- | --- | --- | --- | --- | --- |
| 511 | 6.08E-07 | 2 | 118 | GO:0004175 | endopeptidase activity | 0.00038327 | Pathogen vs Control |
| 365 | 4.16E-06 | 2 | 202 | GO:0008233 | peptidase activity | 0.00130975 | Pathogen vs Control |
| 567 | 1.78E-05 | 2 | 70 | GO:0008237 | metallopeptidase activity | 0.00374152 | Pathogen vs Control |
| 601 | 0.0003543 | 2 | 43 | GO:0004222 | metalloendopeptidase activity | 0.05580265 | Pathogen vs Control |
| 439 | 1.83E-05 | 2 | 118 | GO:0004175 | endopeptidase activity | 0.01151802 | Probiotic vs Control |
| 528 | 2.66E-07 | 2 | 118 | GO:0004175 | endopeptidase activity | 0.00016769 | Probiotic vs Pathogen |
| 349 | 1.10E-05 | 2 | 202 | GO:0008233 | peptidase activity | 0.00345314 | Probiotic vs Pathogen |
| -225 | 0.00042962 | 2 | 322 | GO:0008092 | cytoskeletal protein binding | 0.0902211 | Probiotic vs Pathogen |
